# Heritability and age-dependent changes in genetic variation of telomere length in a wild house sparrow population

**DOI:** 10.1093/evlett/qrae055

**Published:** 2024-11-21

**Authors:** Heung Ying Janet Chik, Aaron Sibma, Maria-Elena Mannarelli, Natalie dos Remedios, Mirre J P Simons, Terry Burke, Hannah L Dugdale, Julia Schroeder

**Affiliations:** Groningen Institute for Evolutionary Life Sciences, University of Groningen, Groningen, The Netherlands; School of Natural Sciences, Macquarie University, Sydney, New Sout Wales, Australia; Ecology and Evolutionary Biology, School of Biosciences, University of Sheffield, Sheffield, United Kingdom; Ecology and Evolutionary Biology, School of Biosciences, University of Sheffield, Sheffield, United Kingdom; School of Biological Sciences, University of East Anglia, Norfolk, United Kingdom; Ecology and Evolutionary Biology, School of Biosciences, University of Sheffield, Sheffield, United Kingdom; School of Social Sciences, University of Auckland, Auckland, New Zealand; Ecology and Evolutionary Biology, School of Biosciences, University of Sheffield, Sheffield, United Kingdom; Ecology and Evolutionary Biology, School of Biosciences, University of Sheffield, Sheffield, United Kingdom; Groningen Institute for Evolutionary Life Sciences, University of Groningen, Groningen, The Netherlands; Ecology and Evolutionary Biology, School of Biosciences, University of Sheffield, Sheffield, United Kingdom; Department of Life Sciences, Imperial College London Silwood Park, Ascot, United Kingdom

**Keywords:** telomere dynamics, heritability, genotype-by-age interaction, quantitative genetics, senescence

## Abstract

Telomere length (TL) and/or its rate of change are popular biomarkers of senescence, as telomere dynamics are linked with survival and lifespan. However, the evolutionary potential of telomere dynamics has received mixed support in natural populations. To better understand how telomere dynamics evolve, it is necessary to quantify genetic variation in TL and how such variation changes with age. Here, we analyzed 2,083 longitudinal samples from 1,225 individuals across 16 years, collected from a wild, insular house sparrow (*Passer domesticus*) population with complete life history and genetic relatedness data. Using a series of “animal” models, we confirmed that TL changes with age, reflecting senescence in this population. We found TL to be repeatable (14.0%, 95% CrI: 9.1%–19.9%) and heritable (12.3%, 95% CrI: 7.5%–18.2%); and detected a genotype-by-age interaction, meaning that genotypes differ in their rate of change of TL, and additive genetic variance increases at older ages. Our findings provide empirical evidence from a wild population that supports hypotheses explaining the evolution of senescence and highlight the importance of telomere dynamics as a key biomarker of body physiology for the evolution of senescence.

## Introduction

How variation in senescence, the decline in body state with age resulting in death (Monaghan et al., 2008), arises is a central question in evolutionary biology. To quantify senescence and study its evolution, telomere dynamics have become a popular biomarker. Telomeres are highly conserved, repeating DNA sequences primarily capping the ends of chromosomes (Meyne et al., 1989). Telomeres are important for maintaining DNA integrity and protecting coding DNA from erosion caused by the lagging strand of linear DNA not being fully replicated, i.e., the end-replication problem (Olovnikov, 1973). Thus, in each cell replication cycle, telomeres shorten (Blackburn, 1991). Telomere shortening can also be accelerated, e.g., by stress exposure (Chatelain et al., 2020; von Zglinicki, 2002), but elongation can also occur through telomerase action and other mechanisms (Biessmann & Mason, 2003; Blackburn et al., 1989). However, telomerase is typically suppressed in adult mammalian or human cells (Blackburn et al., 2015). When telomeres shorten to a critical length, cell division ceases, and the cell enters a state of senescence (Blackburn et al., 2015). The accumulation of senescent cells can result in a decline in tissue function (Campisi, 2005). As such, telomere length (TL) could reflect the intrinsic state of an individual and has become a biomarker for senescence. Indeed, while the specific causal mechanism is still unclear (Simons, 2015), increasing evidence has linked short telomeres and/or telomere shortening to decreased survival and lifespan in natural populations (Eastwood et al., 2019; Heidinger et al., 2012; Wilbourn et al., 2018; Wood & Young, 2019), age-related disease and mortality in humans (Blackburn et al., 2015; Cawthon et al., 2003), and decreased reproductive output (Heidinger et al., 2021). Consequently, TL can be under selection and play a part in the evolution of senescence.

To confirm that TL could evolve and to test the theories explaining the evolution of senescence on TL, one needs to demonstrate the presence of its genetic variance. Estimates for the proportion of additive genetic variance ($V_{a}$) to total phenotypic variance ($V_{t}$)—the heritability—range from 0 to 1 among vertebrate studies (Chik et al., 2022; Dugdale & Richardson, 2018). This variation is partly driven by the choice of statistical methods, as commonly applied methods confound genetic and common environmental effects, resulting in inflated heritability estimates (Chik et al., 2022). Also, the majority of heritability estimates come from human studies and laboratory animals of controlled ages and environments, limiting our ability to deduce the roles of selection and evolution under natural conditions (Wilson et al., 2008). Furthermore, under natural conditions, genotype-by-age interactions (G × A) are likely to occur when genotypes differ in their rates of senescence, resulting in an increase in $V_{a}$ with age (Charmantier et al., 2014).

Such age-related changes in $V_{a}$ can indicate selection patterns and evolutionary processes that give rise to senescence itself (Wilson et al., 2008). Two nonmutually-exclusive evolutionary hypotheses explain the origin of senescence (Maklakov & Chapman, 2019). First, the mutation accumulation hypothesis posits that, due to extrinsic mortality risks, cohorts decline in number and reproductive potential as they age, weakening the selection pressure against deleterious mutations in later life and allowing senescent phenotypes to persist (Medawar, 1952). Second, the antagonistic pleiotropy hypothesis posits that, as population size is larger in younger age classes, pleiotropic mutations that provide benefits in early life but have damaging effects in later life would be selected for (Williams, 1957). Both hypotheses are not mutually exclusive, and both predict G × A, where selection weakens with increasing age, leading to increasing $V_{a}$ in senescing traits, while the antagonistic pleiotropy theory additionally predicts a negative genetic correlation between early-life and late-life trait values (Wilson et al., 2008). However, it is often difficult, and also rarely a main goal, to distinguish between the two theories using a quantitative genetic approach (Wilson et al., 2008). Studies examining G × A in the wild have, however, provided mixed results—significant G × A in fitness-related traits has been found in some species (Chantepie et al., 2015; Charmantier et al., 2006; Wilson et al., 2007) but not others (Brommer et al., 2007, 2010). Thus far, only a few studies have tested for G × A in TL: Bauch et al. (2021) found significant but low heritability in TL shortening in free-living jackdaws (*Corvus monedula*); Pepke et al. (2023) found higher heritability in TL change in wild house sparrows (*Passer domesticus*); while Seeker et al. (2018) and Vedder et al. (2021) did not find support for G × A in captive diary cattle (*Bos taurus*) and wild common terns (*Sterna hirundo*) respectively. Here, we found G × A in TL in a wild, isolated house sparrow population and demonstrated that TL senesces as predicted by evolutionary theory.

## Methods

### Study population and data collection

The house sparrow (*Passer domesticus*) is a gregarious and socially monogamous passerine that readily uses nestboxes and is sedentary in nature with limited movement (De Laet & Summers-Smith, 2007). We collected telomere, life history and pedigree data from a free-living, nestbox-breeding population of house sparrows on Lundy Island (51°10ʹN, 4°40ʹW), 19 km off the coast of Devon, United Kingdom. We have systematically monitored this population since 2000. Owing to the small size of the island and its geographical isolation limiting immigration and emigration (Schroeder et al., 2015), we were able to tag and identify > 99% of all sparrows hatched on Lundy since 2000 with a uniquely numbered metal ring from the British Trust for Ornithology and a unique combination of three color rings. Every year, we recorded all birds breeding in nestboxes, including the identities of the parent of each brood, the offspring identities and hatch dates. This allowed us to determine the exact age of each bird at sampling. A small minority of birds fledged from inaccessible nests, and we captured them with mist nets, both during the breeding season, immediately after they fledged (April to August), and during the following annual winter census visit (November to December). We assumed these birds hatched during the breeding season of that year. Due to the mobile nature of birds, it is typically difficult, if not impossible, to gather such precise age and death estimates in natural populations. Therefore, the above-listed characteristics of our study populations render our life history (e.g., age, birth year) and parentage data unusually precise.

To quantify TL and assign genetic parentage, we collected blood samples repeatedly from individual birds, systematically at two and 12 days of age, during their first winter, and on every subsequent capture. Previous analyses of these data suggested that all birds were equally likely to be caught and sampled (Simons et al., 2015). We stored blood samples in 96% ethanol at room temperature until DNA extraction. In addition, to distinguish the effects of the genetic parents, the environment during incubation, and the environment posthatching, on average, 39% of chicks were cross-fostered at two or three days of age during every breeding season (Winney et al., 2015). All animal procedures were approved by the UK Home Office.

### Telomere extraction and assay

We measured TL using blood samples collected from sparrows after fledging between 2000 and 2015. Assays were conducted from 2014 to 2016, and blood sample storage time ranged from 0 to 15 years. We extracted DNA using an ammonium acetate extraction method (Richardson et al., 2001). Extracted DNA was stored in TE buffer (10 mM Tris, 0.1 mM EDTA) at −20 °C until telomere analysis. Prior to telomere assays, DNA samples were checked for purity by ensuring 260/280 and 260/230 absorbance ratios ≥ 1.8 (Morinha et al., 2020), and their concentrations were measured using a Nanodrop 8000 Spectrophotometer (Thermo Fisher) and normalized to 20–30 ng/µl. Following normalization, we employed a monochrome multiplex quantitative polymerase chain reaction (MMqPCR) method to quantify TL (Cawthon, 2009). For details of the working principles and procedures of the MMqPCR, see (Chik et al., 2024) and the supporting text in the Supplementary Material.

Samples were assigned to qPCR plates using a slicing approach that separates plate effects from biological (e.g., year) effects (van Lieshout et al., 2020; see supporting text). Reactions were run using two machines, a QuantStudio 12K Flex Real-Time PCR System (Thermo Fisher Scientific, five plates) and a StepOnePlus (Applied Biosystems, 77 plates), but machine identity was not correlated with the final T/S ratios (Sibma, 2021). Plates were run by two technicians (MEM ran 52 plates and NdR ran 30 plates). As reported in (Chik et al., 2024), MMqPCR procedures were verified by the following metrics: The mean qPCR amplification efficiencies for telomeres and the reference gene were 89.2% (SD = 8.1%, range = 70.7%–110%) and 88.0% (SD = 6.8%, range = 70.7%–105%) respectively; the intra-plate repeatability of TL was 95.7% (SE = 0.2%, *N* = 4536 observations from 2162 samples); the inter-plate repeatability of Ct for telomeres, calculated from serially diluted standard samples across plates, was 98.3% (SE = 0.4%, *N* = 126 from 63 samples), and that of Ct for the reference gene was 99.0% (SE = 0.2%); and finally, the inter-extraction repeatability TL was 52.3% (SE = 6.9%, *N* = 232 observations from 48 samples). Our final full dataset consisted of 2,083 TL measurements from 1,225 birds, 476 of which have at least two TL observations. Further telomere dataset summaries are provided in Supplementary Tables S1 and S2.

### Genetic pedigree construction

We used up to 23 house sparrow microsatellite markers (Dawson et al., 2012) to construct a genetic pedigree for individuals born 1995–2019, using Cervus 3.0 (Marshall et al., 1998). In brief, we first ran an identity analysis to resolve potential field sampling and lab errors, then ran a maternal analysis to confirm the genetic mother, and finally, a biparental analysis to assign the genetic father (Schroeder et al., 2019). We then pruned the pedigree to include only informative individuals, i.e., individuals with TL measurements and those linking these individuals. The pruned pedigree consisted of 1,321 birds, with 1,196 assigned maternities, 1,197 assigned paternities, and a maximum pedigree depth of 16 generations (Supplementary Figure S1). Pedigree statistics were calculated using *pedantics* 1.7 (Morrissey & Wilson, 2010) and are summarized in Supplementary Table S3.

### Statistical analysis

All analyses were carried out in R 4.0.3 (R Core Team, 2021). Regression models were built using the Bayesian package *MCMCglmm* 2.29 (Hadfield, 2010). For each model, we adjusted the number of iterations, burn-in, and thinning interval such that convergence was reached based on the following criteria: visual inspection of posterior trace plots showed no distinguishable trend, autocorrelation was lower than 0.1, the effective sample size was greater than 1,000.

### Age-dependent changes in TL

To first verify that TL varies with age, we built a linear mixed model (LMM, Model 1), where the T/S ratio was the response variable, assuming a Gaussian residual distribution. Log-transforming TL did not provide a better model fit. To examine individual senescence patterns, we separated within-individual and between-individual effects by fitting both the age mean-centered within each individual (WiAge, in years) and the mean age of each individual (BtAge, in years) as explanatory variables (van de Pol & Wright, 2009). Following Fay et al. (2022), we also tested for a nonlinear relationship between TL and between- and within-individual age. First, we fitted a model with squared terms of BtAge and WiAge, which assumed that the within-individual age effect was a relative process dependent upon the mean age of each individual. Second, we also fitted a model where we quantified within-individual quadratic age effects as the difference between the actual age squared and BtAge squared (Equation 3b, Fay et al., 2022). This model assumes the within-individual aging process was absolute and did not depend upon the mean individual age. Fitting age mean-centered over the whole population (McAge, in years), or age as a factor did not provide a better model fit. To test for differences in TL between males and females, we fitted sex as a two-level fixed factor. As TL decreases with sample storage time nonlinearly in our dataset (Sibma, 2021), we fitted the duration for which the blood sample was stored before DNA extraction (Blood Age, in years), the duration for which the extracted DNA was stored before telomere assay (DNA Age, in years), and their squared terms. As TL differed between the two technicians (Wilcoxon rank sum test: *W* = 233,714, *p* < 0.001), we also added technician ID as a two-level fixed factor. Finally, as random variables, we fitted individual bird ID to account for variation in TL among birds, plate ID and row ID to account for technical variance among qPCR plates and among row positions on each plate (Eisenberg et al., 2015). Because within each individual, TL was measured on average 1.5 times per year, the within-individual term essentially captures the within-individual changes in TL from year to year. Therefore, we did not fit sampling year into the model. We used default (flat improper, weakly informative) priors for fixed effects and uninformative inverse-Wishart priors (*V* = 1, ν = 0.002) for random effects. The model remained robust when another relevant prior (parameter-expanded prior: *V* = 1, ν = 0.002, *αμ* = 0, *αV* = 1,000) was used (Supplementary Table S4).

The TL–age relationship was linear in our data, and sex had no effect (see *Results* section). Hence, we removed the quadratic terms of WiAge, BtAge, and sex from the fixed effects structure in subsequent analysis. The removal of these terms did not impair model fit (ΔDIC = −4.515). As a significant difference between the within- and among-individual slopes could lead to a biased estimation of the individual variances in the random effects structure (Westneat et al., 2020), we tested for this difference by further fitting an LMM (Model 2), where WiAge was replaced with untransformed age (in years):


$$\begin{array}{lllll} & TL\;∼\;Untransformed\;age+BtAge+BloodAge\; & +\;BloodAge^{2}+DNAAge\; & +\;DNAAge^{2}+TechnicianID\; & +\left(1\;|\;BirdID\right)+\left(1\;|\;Plate\right)+(1|Row) \end{array}$$ (i)

In this model, the untransformed age effect represents the within-individual slope, while the BtAge effect represents the difference between the within- and between-individual slope (van de Pol & Wright, 2009). The two slopes were statistically significantly different from each other (posterior mode for BtAge = 0.063, 95% CrI = 0.027–0.103).

(1) *TL repeatability and heritability*

To estimate the additive genetic ($V_{a}$) and permanent environmental variance ($V_{pe}$) in TL, we expanded Model 1 into a series of “animal” models with sequentially increasing random variables. Using this approach allows us to examine how $V_{a}$ and heritability (see same paragraph below) estimates change when we add in more potentially confounding environmental variables (Sparks et al., 2020). While storage time was correlated with cohort effects (*r* = −0.73 for BloodAge and *r* = −0.28 for DNAAge), we opted to retain storage time in the fixed effect structure, as we aimed to estimate cohort variance after these methodological effects were accounted for (de Villemereuil et al., 2018). In Model 3, we fitted an individual “animal” term linked to the pruned pedigree, in addition to the individual “bird ID” term, allowing the separation of individual variance into genetic and permanent environmental components. In Models 4 and 5, we added the identity of the rearing mother and father, respectively, to estimate the variance due to nongenetic parental effects during rearing. In Model 6, we added the year of capture to account for potential yearly environmental stress effects on TL. Finally, in Model 7, we added the year in which the individual was born (cohort) to estimate the effect of the hatching year. To check that storage time effects did not influence cohort variance estimate, we also reran Model 7 without BloodAge and DNAAge terms. This reduced model returned similar cohort variance as the unaltered model (*V* = 0.000, 95% CI = 0.000–0.003; cf results below, Figure 2; Supplementary Table S10). For each model, we calculated individual repeatability as ($V_{a}$ + $V_{pe}$)/$V_{t}$, and heritability as $V_{a}$/$V_{t}$, where $V_{t}$ is the sum of all variance components and residual variance, except those of plate ID and row ID, as these technical variances are biologically irrelevant. We further calculated the variance explained by the fixed effects WiAge and BtAge, as variances explained by random effects are conditioned on fixed effects, and hence, not including fixed effect variation in calculating $V_{t}$ could lead to underestimation of $V_{t}$ and overestimation of repeatability and heritability (de Villemereuil et al., 2018). However, both fixed effects explained minimal variance (< 0.002), and therefore we did not include them in the final calculation of $V_{t}$. In all “animal” models, we used default priors for fixed effects, parameter-expanded priors (*V* = 1, *ν* = 1, *αμ* = 0, *αV* = 1,000) for random effects, as they improve mixing at the parameter space boundary (Hadfield, 2019), and models using inverse-Wishart priors did not converge. We used inverse-Wishart priors for residuals.

(2) *Individual variation in the rate of telomere shortening*

We tested whether individuals differ in their rates of telomere shortening (individual-by-age interaction, or I × A), as such variation would allow scope for G × A. To test for I × A, we fitted a random regression model (RRM) with TL as the response variable. For the fixed effect structure, we fitted McAge and retained all storage variables and technician ID from the previous models. For the random effects structure, we modeled individual variation in TL as a function of age, in addition to effects of the year of capture, plate 1D and row ID. We excluded identities of the rearing parents and cohort in the random effect structure, as these variables explained negligible variances (see *Results* section). Because heterogeneity in the residuals could lead to false positives and overestimation of phenotypic variances (Ramakers et al., 2020), we fitted a heterogenous residual structure by pooling TL measurements into four stages: (1) “juvenile” (age 0); (2) “young” (ages 1 and 2); (3) “middle age” (ages 3 and 4); and (4) “old” (ages 5 or above), estimating one residual variance for each age class, and the among-age-class covariances. Models attempting to estimate one residual variance for each age (0–7) did not converge, while models with a homogenous residual structure (i.e., estimating one residual variance value) returned similar results but fitted the data less well (Supplementary Table S5, ΔDIC = −236). The final model equation for the RRM was thus:


$$\begin{array}{lllll} TL= & \;\mu +\;McAge+BloodAge+BloodAge^{2}+DNAAge\; & +\;DNAAge^{2}+\;TechnicianID+f\left(ID,\;age^{*}\right)\; & +\;Capture\;year+Plate\; & +Row+\;f\left(\varepsilon ,AgeClass\right) \end{array}$$ (ii)

where $f\left(ID,\;age^{*}\right)$ is the random regression function for individuals. For this random effect, we used Legendre polynomials following (Chantepie et al., 2015) and (Kirkpatrick et al., 1990), where age is rescaled to a range of −1 to 1 (from 0 to 7) by:


$$\begin{array}{l} age_{i}^{*}=\;-1+\;\frac{2}{age_{max}-\;age_{min}}\;\left(age_{i}-\;age_{min}\right) \end{array}$$ (iii)

where $age_{i}^{*}$ is the rescaled age, $age_{i}$ is the original age, $age_{max}$ is the maximum age recorded in the whole dataset, and $age_{min}$ is the minimum age recorded in the whole dataset. While the choice of the class of orthogonal polynomials does not affect the estimation of inter-age covariances over the age range in which the data were collected, it would affect extrapolation outside of this range (Kirkpatrick et al., 1990). As we only found a linear TL–age relationship within individuals, we only fitted the first two Legendre polynomials:


$$\begin{array}{l} {\;\varphi }_{0}=\;\frac{1}{\sqrt{2}} \end{array}$$ (iv)

$$\begin{array}{l} \varphi_{1}=\;\;\;\sqrt{\frac{3}{2}}x \end{array}$$ (v)

We used inverse-Wishart priors to estimate both random and residual structures.

(3) *Changes in additive genetic variance in relative TL*

To assess whether the rate of telomere shortening had a genetic basis, we built a random regression animal model (RRAM) from the RRM above, where we partitioned the individual variation in the TL–age slope into genetic and permanent environmental components by fitting an “animal” random effect term linked to the genetic pedigree. We retained the same fixed effect structure, additional random variables, and residual structure as for the RRM above. Thus, the model equation was:


$$\begin{array}{llllll} TL= & \;\mu +\;McAge+BloodAge+BloodAge^{2}+DNAAge\; & +\;DNAAge^{2}+\;TechnicianID\; & +f\left(a,\;age^{*}\right)+\;f\left(\;pe,\;age^{*}\right)\; & +\;Capture\;year+Plate+Row\; & +\;f\left(\varepsilon ,AgeClass\right) \end{array}$$ (vi)

where $f\left(a,\;age^{*}\right)$ represents the random regression function for the additive genetic effect and $f\left(\;pe,\;age^{*}\right)$ that of the permanent environmental effect. This RRAM successfully converged, with the inclusion of the “animal” term improving model fit (ΔDIC = −25), but estimated similar genetic and permanent environmental variances (Table 1), which when summed exceeded the phenotypic slope variation. This could be due to MCMC chains experiencing difficulties in allocating small permanent environmental variation. Therefore, we ran a second RRAM including the “animal” term only. This second RRAM returned similar additive genetic variance and covariance estimates (Table 2) and had better fit than the first RRAM (ΔDIC = −24.7). This further suggested that the presence of genetic variance in the rate of change of TL was statistically supported, while that of permanent environmental variance was not. To examine the changes in $V_{a}$ with age, we transformed the estimates of the additive genetic coefficients from the second RRAM by

**Table 1.** Summary of the random regression “animal” model (RRAM) testing for additive genetic (“Animal” term) and permanent environmental (“Bird ID” term) variation in the mean telomere length (TL) and rate of TL change with age, among the Lundy house sparrows sampled in 2000–2015.

	Post. mode	95% CrI	Effective sample size	pMCMC
*Fixed effects*				
(Intercept)	1.664	**1.404–1.900**	**13,500**	**<0.0001**
McAge	−0.002	−0.056–0.046	13,500	0.825
**BloodAge**	**−0.097**	**−0.149** to −**0.064**	**13,439**	**<0.0001**
**BloodAge** ^**2**^	**0.004**	**0.001–0.006**	**13,500**	**0.010**
DNAAge	0.017	−0.030–0.073	13,500	0.470
**DNAAge** ^**2**^	**−0.007**	**−0.012** to −**0.003**	**13,500**	**0.001**
Technician (B)	0.015	−0.122–0.140	13,500	0.877
*Random effects*				
Animal				
Intercept	0.060	0.041–0.094	10,941	
Slope	0.074	0.052–0.127	10,672	
**Intercept:Slope**	**0.034**	**0.014–0.071**	**10,537**	
BirdID				
Intercept	0.055	0.038–0.084	9908	
Slope	0.076	0.051–0.122	10,817	
**Intercept:Slope**	**0.033**	**0.016–0.068**	**10,064**	
Year	0.029	0.012–0.080	13,500	
Plate	0.036	0.023–0.054	13,500	
Row	0.002	0.000–0.008	13,167	
Residuals				
Juvenile	0.147	0.127–0.171	13,500	
Young	0.170	0.152–0.191	13,500	
MiddleAge	0.151	0.117–0.197	6,897	
Old	0.165	0.109–0.252	2,177	
Juv:Young	−0.002	−0.042–0.045	4,361	
Juv:Mid	0.002	−0.041–0.040	4,266	
Juv:Old	0.001	−0.046–0.045	3,330	
Young:Mid	0.003	−0.045–0.044	3,461	
Young:Old	0.001	−0.048–0.051	2,697	
Mid:Old	0.004	−0.045–0.050	3,062	

*Note*. Statistically significant fixed effects and covariances are in bold. Post. mode = posterior mode, 95% CrI = 95% credible interval; pMCMC = MCMC *p*-value. McAge = population mean-centered age; Blood Age = storage time as blood sample (in years); DNA Age = storage time as DNA sample (in years); Technician (*N* = 2; contrast = A); Animal = genetic variances and covariances; BirdID = permanent environmental variances and covariances; Year = Year of capture; Plate = qPCR plate ID; Row = Row ID on qPCR plate. For (pooled) age classes in the residuals: Juvenile/Juv = 0; Young = 1–2; MiddleAge/Mid = 3–4; Old = 5+. DIC = 2422.

**Table 2.** Summary of the RRAM testing for only additive genetic (“Animal” term) variation in the mean TL and rate of TL change with age among the Lundy house sparrows sampled in 2000–2015.

	Post. mode	95% CrI	Effective sample size	pMCMC
*Fixed effects*				
(Intercept)	1.700	1.393–1.886	13,238	<0.0001
McAge	0.002	−0.051–0.041	12,823	0.896
**Blood Age**	**−0.101**	**−0.143** to −**0.060**	**12,739**	**<0.0001**
**Blood Age** ^**2**^	**0.003**	**0.001–0.006**	**16,133**	**0.017**
DNA Age	0.023	−0.033–0.069	13,500	0.480
**DNA Age** ^**2**^	**−0.008**	**−0.012** to −**0.003**	**14,066**	**0.002**
Technician(B)	0.020	−0.109–0.149	13,500	0.751
*Random effects*				
Animal				
Intercept	0.056	0.042–0.090	12,174	
Slope	0.076	0.048–0.113	11,224	
**Intercept:Slope**	**0.024**	**0.009–0.057**	**11,021**	
Year	0.028	0.012–0.081	13,876	
Plate	0.034	0.024–0.054	13,500	
Row	0.001	0.000–0.008	13,941	
Residuals				
Juvenile	0.173	0.153–0.198	13,500	
Young	0.182	0.164–0.203	13,500	
MiddleAge	0.188	0.150–0.228	9,115	
Old	0.147	0.106–0.241	2,306	
Juv:Young	0.006	−0.052–0.051	3,512	
Juv:Mid	0.000	−0.052–0.053	3,165	
Juv:Old	−0.004	−0.050–0.050	2,804	
Young:Mid	−0.001	−0.053–0.057	2,873	
Young:Old	−0.002	−0.049–0.053	2,456	
Mid:Old	0.001	−0.052–0.052	2,746	

*Note*. Statistically significant fixed effects and covariances are in bold. Post. mode = posterior mode, 95% CrI = 95% credible interval; pMCMC = MCMC *p*-value. McAge = population mean-centered age; Blood Age = storage time as blood sample (in years); DNA Age = storage time as DNA sample (in years); Technician (*N* = 2; contrast = A); BirdID = unique individual identifier; Year = Year of capture; Plate = qPCR plate ID; Row = Row ID on qPCR plate. For (pooled) age classes in the residuals: Juvenile/Juv = 0; Young = 1–2; MiddleAge/Mid = 3–4; Old = 5+. DIC = 2397.


$$\begin{array}{l} G=\;\Phi C\Phi^{T} \end{array}$$ (vii)

where G is the inter-age additive genetic variance–covariance matrix, C is the RRAM coefficient matrix, and Φ is a matrix defined such that $\Phi_{ij}=\;{\;\varphi \;}_{0}\left(age{*}_{i}\right)$ (Kirkpatrick et al., 1990).

To verify the RRAM, we further fitted a “character-state model,” where age-specific TL measurements were treated as correlated subtraits. We first corrected TL measurements by fitting a mixed model with BloodAge, DNAAge, and their square terms, plus technician ID as fixed predictors, and plate ID as a random predictor. The residual TL values were then pooled into the above four age classes. When an individual was sampled more than once within each age class, we took the mean of these TL measurements. We then fitted a multivariate animal model in MCMCglmm, where measurements from the four stages were fitted as multivariate response variables, and the animal and BirdID terms as random variables, allowing genetic variance and covariances among age classes to be estimated. Similar to our approach with the RRAM, we reran this character state model using the BirdID term only and found statistical support for the inclusion of the animal term (ΔDIC = −91, Supplementary Table S6).

## Results

**Figure 1.**
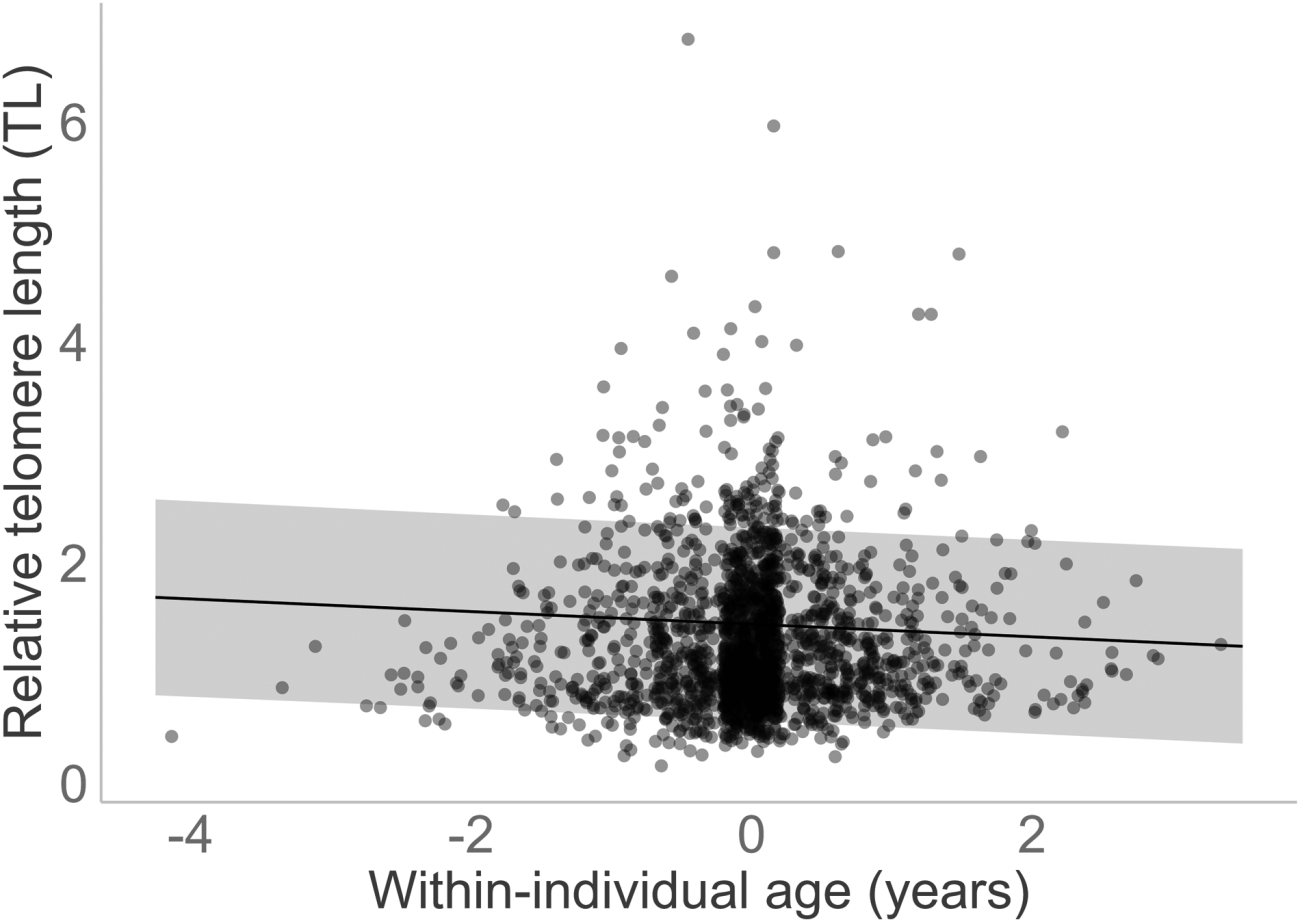
Linear relationship between relative telomere length (TL) and within-individual mean-centered age estimated from the Lundy house sparrows sampled in 2000–2015. The black line indicates the predicted relationship, while the shaded area represents the 95% CrI. Black circles (jittered along the *x*-axis) represent the raw data.

We verified that telomeres shortened as individuals aged, as TL was negatively correlated with within-individual age but not across birds of different ages (Figure 1; Supplementary Table S7). This within-individual age effect was linear, as a quadratic effect did not reach significance, irrespective of whether we assumed the within-individual aging process to be dependent on the mean individual age or not (Supplementary Tables S8 and S9). TL did not differ between the sexes (Supplementary Table S7). TL was influenced by storage effects, specifically, the duration of time that the sample was stored as (a) blood before DNA extraction and (b) DNA before TL measurement (Supplementary Table S7).

**Figure 2.**
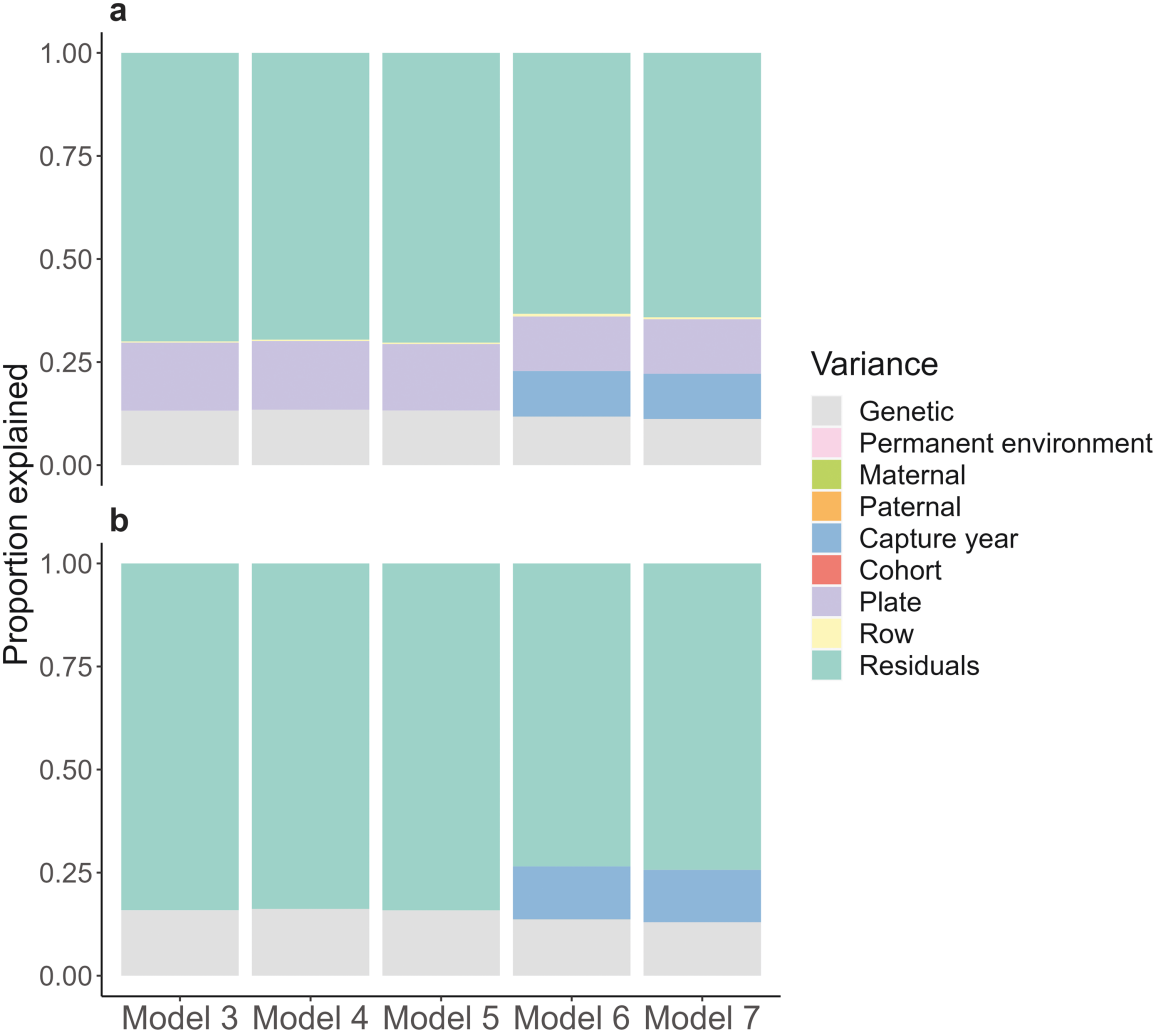
Variance components from a series of “animal” models (Models 3–7) to estimate sources of variation in TL in the Lundy house sparrow population sampled in 2000–2015: (A) the proportions of all fitted random variables, and (B) proportions of biologically relevant random variables only, i.e., excluding plate and row variances.

From the “animal” models, TL showed moderate repeatability and heritability (Model 7, individual repeatability = 14.0% (95% CrI: 9.1%–19.9%), heritability = 12.3% (95% CrI: 7.5%–18.2%), Supplementary Table S10; Figure 2). Little variation in TL was explained by the identities of the rearing parents, and by cohort, but capture year accounted for 12.6% (95% CrI 5.7%–29.7%; Model 7, Supplementary Table S10; Figure 2) of the phenotypic variance.

The rates of how fast telomeres shortened with age differed among individuals, evidenced by the statistically significant variance in their intercepts and slopes in our RRM (Table 3). Individuals that initially had a longer TL showed a slower rate of telomere shortening, indicated by a statistically significant positive covariance between the individual intercept and slope (less negative slope; Table 3).

**Table 3.** Summary of the random regression model testing for individual variation in the mean TL and rate of TL change with age among Lundy house sparrows sampled in 2000–2015.

	Post. mode	95% CrI	Effective sample size	pMCMC
*Fixed effects*				
(Intercept)	1.596	1.342–1.811	13,500	<0.0001
McAge	−0.008	−0.033–0.017	13,099	0.553
**Blood Age**	**−0.098**	**−0.146** to −**0.063**	**13,500**	**<0.0001**
**Blood Age** ^**2**^	**0.003**	**0.001–0.006**	**13,500**	**0.009**
DNA Age	0.021	−0.030–0.072	12,733	0.438
**DNA Age** ^**2**^	**−0.007**	**−0.012** to −**0.003**	**12,796**	**0.002**
Technician (B)	0.012	−0.116–0.148	13,500	0.781
*Random effects*				
BirdID				
Intercept	0.058	0.039–0.084	11,570	
Slope	0.080	0.051–0.120	12,704	
**Intercept: Slope**	**0.023**	**0.010–0.058**	**11,424**	
Year	0.033	0.013–0.088	13,720	
Plate	0.036	0.024–0.054	13,500	
Row	0.001	0.000–0.008	13,500	
Residuals				
Juvenile	0.161	0.140–0.188	13,343	
Young	0.183	0.161–0.201	13,500	
MiddleAge	0.170	0.140–0.222	7,990	
Old	0.157	0.104–0.238	2,245	
Juv:Young	−0.002	−0.048–0.048	3,795	
Juv:Mid	−0.003	−0.050–0.049	3,456	
Juv:Old	0.004	−0.047–0.045	3,326	
Young:Mid	0.008	−0.053–0.052	3,044	
Young:Old	−0.002	−0.048–0.050	2,739	
Mid:Old	−0.005	−0.047–0.051	2,930	

*Note*. Statistically significant fixed effects and covariances are in bold. Post. mode = posterior mode, 95% CrI = 95% credible interval; pMCMC = MCMC *p*-value. McAge = population mean-centered age; Blood Age = storage time as blood sample (in years); DNA Age = storage time as DNA sample (in years); Technician (*N* = 2; contrast = A); BirdID = unique individual identifier; Year = Year of capture; Plate = qPCR plate ID; Row = Row ID on qPCR plate. For (pooled) age classes in the residuals: Juvenile/Juv = 0; Young = 1–2; MiddleAge/Mid = 3–4; Old = 5+. DIC = 2447.

**Figure 3.**
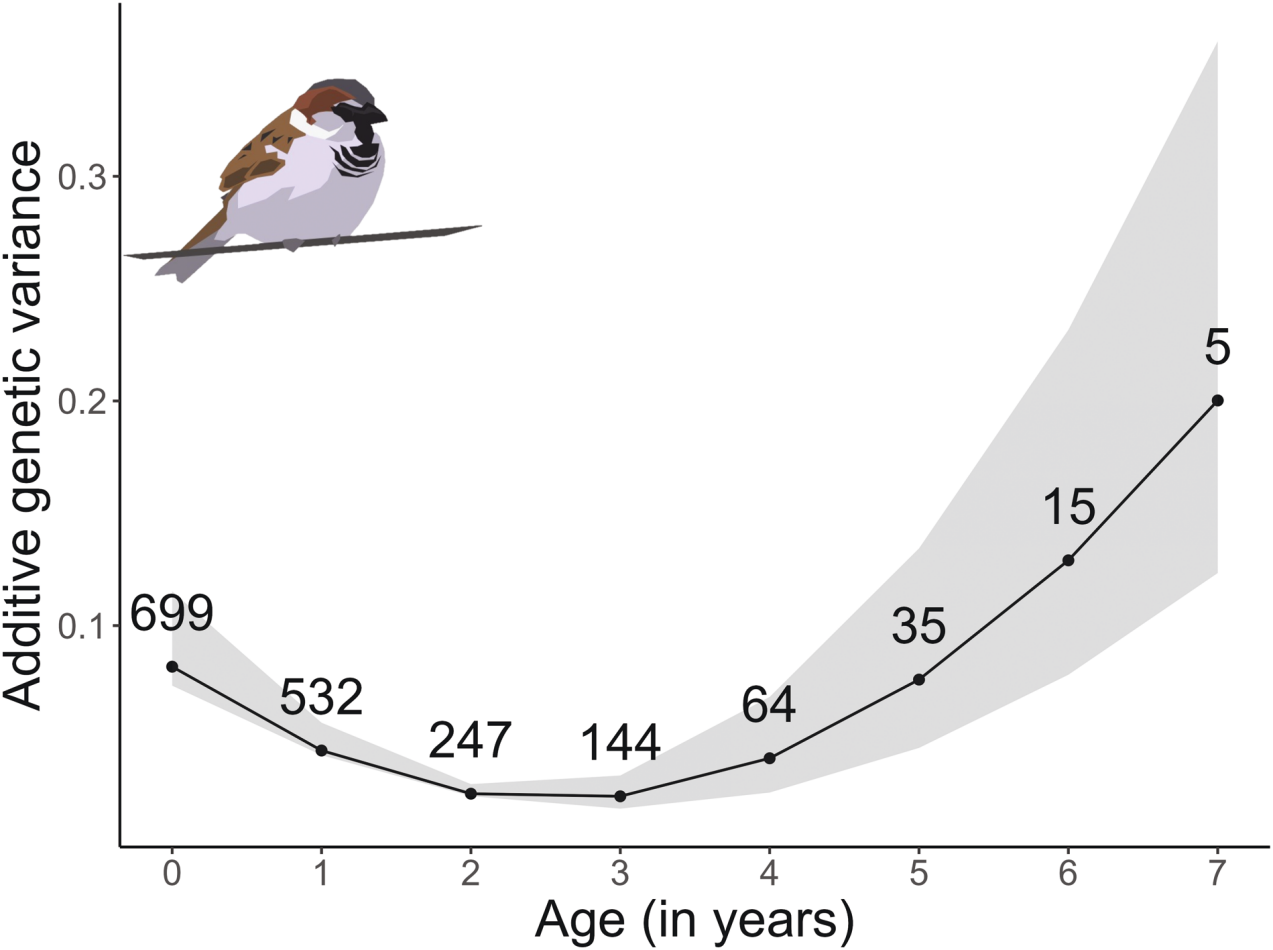
The quadratic relationship between the additive genetic variance of TL and age in Lundy house sparrows sampled in 2000–2015. Black dots represent point estimates of additive genetic variance for each age class (0–7), and the shaded area around each dot represents the 95% CrI of the respective point estimate. Numbers above each point estimate indicate the number of birds belonging to that age class. Sparrow illustration image credit: J. Dunning.

We detected a G × A effect in TL, indicated by the random regression “animal” model, where both TL and the rate of TL change had a statistically significant additive genetic component (Table 1). This means there is a genetic link between having longer telomeres and slower telomere shortening, detected by the significant genetic covariance between the intercept and the slope (Table 1). Finally, the inter-age additive genetic matrix showed that $V_{a}$ decreased up to 3 years of age and then increased at later ages (Figure 3; Supplementary Table S11). This pattern was confirmed by a “character-state animal model,” where genetic variances in TL classified into life stages were high at “juvenile” stage, lowest at “young,” and increased through “middle age” and “old” ages (Supplementary Table S12).

## Discussion

In this study, we investigated genetic and environmental sources of variation in telomere dynamics in a free-living passerine and provided evidence for individuals differing in the rate of telomere shortening and that this shortening has a genetic component indicative of G × A. These results support both the mutation accumulation and antagonistic pleiotropy hypotheses that explain the evolution of senescence.

We also showed that TL undergoes senescence in adult wild birds, in line with reports in other natural systems (Bichet et al., 2020; Froy et al., 2021; Remot et al., 2021). Beyond the rapid growth period during early life, when telomeres shorten rapidly, telomeres in adults generally decline at a slower but steady rate, chiefly due to the accumulation of environment-induced damage and the general suppression of lengthening mechanisms (Monaghan & Ozanne, 2018). However, in our system, TL for older birds was, on average, similar to that for younger ones, likely because old individuals with short telomeres had not survived and were thus not sampled, resulting in the leveling off of the between-individual relationship between age and TL. This selective disappearance was further supported by our finding that TL was positively correlated with survival independent of age (Chik et al., 2024).

Our heritability of 12.3% is similar to that found in another house sparrow population (Pepke et al., 2023) but is low compared to the global average among vertebrates (44.9%; Chik et al., 2022), and to some bird species: 99% in zebra finches (*Taeniopygia guttata*, Atema et al., 2015), 81% in tree swallows (*Tachycineta bicolor*, Belmaker et al., 2019), 77% in jackdaws (*Corvus monedula*, Bauch et al., 2021), 65% in common terns (*Sterna hirundo*, Vedder et al., 2021), and 48% in great reed warblers (*Acrocephalus arundinaceus*, Asghar et al., 2015). However, it is higher than two other wild bird populations: 3.1%–8.0% in Seychelles warblers (*Acrocephalus sechellensis*, Sparks et al., 2020), and 3.8% in white-throated dippers (*Cinclus cinclus*, Becker et al., 2015). Such inconsistency among studies could have a biological explanation, for example being under stronger selection pressure, which reduces genetic variation. However, TL heritability estimates are also influenced by the laboratory assay used to estimate TL, the statistical methods used to estimate heritability, and potentially the age at sampling (Chik et al., 2022), all of which differed between these studies. With increasing age TL is expected to become less heritable as it becomes increasingly dependent on the environment—e.g., oxidative stress and various toxins can accelerate telomere attrition (Monaghan & Ozanne, 2018) and reduce the activity of telomerase, a major telomere lengthening mechanism (Fernandes et al., 2021). As such, in contrast to this study on adult TL, early life TL (Asghar et al., 2015; Bauch et al., 2021; Belmaker et al., 2019) could exhibit higher heritability, as the relative contribution from genetic differences would be higher than the environmental differences at this stage (Dugdale & Richardson, 2018; Pepke et al., 2022). Parallel to this, TL measurement by qPCR, compared to other methods such as terminal restriction fragment methods, introduces higher measurement error, which could also be a cause of higher heritability estimates in studies using the latter method, e.g., (Bauch et al., 2021; Vedder et al., 2021).

Annual stochasticity, e.g., environmental factors that could induce stress, explained a relatively considerable proportion of variance of 11%. In contrast, the identity of the rearing parents did not explain variation in TL, despite better parental care or foster parental quality being associated with longer offspring telomeres in other species (Enokido et al., 2014; Viblanc et al., 2020). Cohort did not explain much variation in adult TL. In our study population, early environmental effects on juvenile TL, if present, may, therefore, not carry over past the developmental stage and into adulthood. Storage time also had a significant effect on TL, in line with previous experimental findings in this population (Sibma, 2021). Blood and DNA storage conditions influence TL measurement—for example, TL varies with blood storage time (Precioso et al., 2022), storage methods (Reichert et al., 2017), DNA concentration (Dagnall et al., 2017), and DNA buffer choice (Eastwood et al., 2018). We were able to partially correct for these effects by including them in our models; yet nevertheless, we encourage researchers to be mindful of the storage protocols used in telomere studies, and we stress the importance of systematic investigation into the effects of storage conditions on TL.

We provided evidence that the rate of change in TL is partially genetically determined, which is expected, as telomere dynamics are complex and influenced by the combined action of many genes (CARDIoGRAM consortium et al., 2013; Pepke et al., 2022). Though Bayesian model variance estimation presented some problems, we believe there is still statistical support for the presence of this G × A interaction. Much remains unknown about telomere maintenance and repair mechanisms, such as the expression of telomerase. While it is evident that this varies vastly across taxa (Karkkainen et al., 2021), we do not know much about how increased antioxidant capacity reduces telomere loss (Monaghan & Ozanne, 2018). Our results emphasize the importance of examining the genetic and environmental influences on these mechanisms and, on an evolutionary level, the importance of understanding whether the rate of telomere shortening is genetically associated with fitness, as this would mean that there could be selection acting on telomere dynamics.

Testing for G × A allowed us to study changes in genetic variation across ages. $V_{a}$ in TL increased from the age of 3 years, in agreement with both the mutation accumulation and antagonistic pleiotropy theories of senescence (Medawar, 1952; Williams, 1957). Both theories are not mutually exclusive and assume that selection pressures weaken at older ages, allowing suboptimal genotypes and, thus greater genetic variation to remain in the population. While the increase of $V_{a}$ at older ages could also be a statistical artifact of fitting second-order Legendre polynomials, our additional analyses (Supplementary Material) provided similar results, supporting this genetic pattern. We also discovered negative genetic covariance in TL between early and late age classes, evidence for antagonistic pleiotropy (Rose, 1990; Wilson et al., 2008). However, as these negative correlations were not observed throughout all early-late age-class pairs and were also not present in the character state model, this interpretation should be made with caution.

We detected a decrease in $V_{a}\;$ between ages 0 and 2–3 years, contrary to an expected uniform increase in genetic variation in fitness-related traits undergoing senescence (Charmantier et al., 2014). There are two plausible explanations for this observed pattern. The first possibility is that certain genotypes lead to telomere lengthening, and that opposing aging trajectories in TL intersect in mid-life, causing higher genetic variance in both early and late life (Charmantier et al., 2014). However, we consider this explanation unlikely, as telomere lengthening currently lacks support in birds (but see (Pepke et al., 2023; Spurgin et al., 2018; also, e.g., Fairlie et al., 2016; van Lieshout et al., 2019 in mammals). Furthermore, lengthening could easily be masked by methodological effects such as measurement error (van Lieshout et al., 2019), leukocyte composition changes, and storage time effects, which significantly influenced TL in our dataset. It is more likely that the decrease in $V_{a}$ during early life pertained to mortality risks. In the Lundy house sparrows, mortality is higher in both early (0–1 years) and later ages (5–7 years) (Chik et al., 2024; Simons et al., 2019). In addition, independent of age, TL is positively linked with survival (Chik et al., 2024). These two findings together suggest a genetic bottleneck leading up to age 2, where young birds with longer TL survive to breeding ages, leading to lower genetic variation in TL around that age.

To better understand how selection shapes genetic variation in TL, an important step would be to examine the links between telomere dynamics and fitness. There is evidence that TL is indeed positively associated with reproductive success in the Lundy house sparrows (Chik et al., 2024), supporting that the G × A patterns observed here could be a result of selection, and thus stressing the significance of telomere dynamics when studying the evolution of senescence.

## Supplementary material

Supplementary material is available online at *Evolution Letters*.

qrae055_suppl_Supplementary_Tables_S1-S12_Figure_S1

## Data Availability

Datasets and R code used in this study are publicly available at: https://doi.org/10.6084/m9.figshare.26517574.
