# Supplementary material for: Heritability and age-dependent changes in genetic variation of telomere length in a wild house sparrow population
Source: Evol Lett. 2024 Nov 21;9(2):209–20. doi: 10.1093/evlett/qrae055 (PMC11968191; doi:10.1093/evlett/qrae055)
Supplement: qrae055_suppl_Supplementary_Tables_S1-S12_Figure_S1 [file qrae055_suppl_supplementary_tables_s1-s12_figure_s1.docx]

**Supporting Information**

**Telomere length quantification using monochrome multiplex quantitative PCR (MMqPCR).**

In this study we measured telomere length in house sparrow blood samples using MMqPCR. In brief, qPCR measures signals of telomeres (T) and those of a single-copy reference gene (S), and quantifies telomere length (TL) as the T/S ratio. Unlike conventional singleplex qPCR, where T and S amplification are conducted in separate wells (Cawthon, 2002), MMqPCR allows T and S signals to be obtained from the same reaction within a single well, thus eliminating error due to differences in the amount of DNA pipetted (Cawthon, 2009). Multiplexing is achieved by using specially designed primers that significantly raise the melting temperature of the S sequence, and by employing a special temperature profile: During earlier, low temperature cycles, cycle threshold (Ct) values of the more abundant telomeres are obtained, when the S signal is still at baseline; during later cycles, the temperature is raised, well above the melting temperature of the telomeric sequences, such that Ct values of the S sequence can be obtained while the T signal goes to baseline. We used the primers telg and telc to prime telomere sequences (Cawthon, 2009), and GAPDH, primed by GAPDH-F (5’- CGGCGGCGGGCGGCGCGGGCTGGGCGGAGC-CAGCCAAGTACGATGACAT - 3’) and GAPDH-R (5’ – GCCCGGCCCGCCGCGCC-CGTCCCGCCGCCATCAGCAGCAGCCTTCA - 3’) as the single-copy reference gene. The GC-clamps in GAPDH-F and GAPDH-R at the 5’ end raise the melting temperature of the S sequence to achieve multiplexing.

We loaded each well with 1.5 μl normalized DNA, 10 μl SYBR Select Master Mix (Applied Biosystems) and each of the four primers at 0.9 μM, totaling 20 μl of reaction mixture. We prepared additionally a series of standard reaction mixtures on each plate using a reference sample diluted to 0.3125, 1.25, 5, 20 and 80 ng/μl, as well as two control wells where DNA was absent. The standard samples allowed the construction of a standard curve to calculate T and S product contents in the reaction mixtures. We ran all qPCR reactions in duplicate in adjacent wells. To avoid confounding biological or environmental effects e.g. cohort, with plate effects during qPCR, we used a slicing approach (van Lieshout et al., 2020): We divided samples from each year into thirds (‘slices’), and allocated them such that plates contained three slices, with one overlapping slice. As a result, recently and formerly collected samples were analysed together on the same plate, and plate effects could then be separated from sampling year and storage time effects. The reactions were run with the following steps: 95°C for 15 min; 2 cycles of 94°C for 15s and 49°C for 15s; 32 cycles of 94°C for 15s, 62°C for 10s and 74°C for 15s with signal acquisition, 84°C for 10s and 86°C for 15s with signal acquisition.

Following MMqPCR, we derived T/S ratios from each reaction using a visual thresholding method: First, we determined background T and S florescent signals as the mean signal values of the earliest cycles before the exponential phase, and subtracted these background values from the raw signals. Second, we plotted log-transformed signal values against cycle number, and visually determined the threshold signal value for both T and S products as the value at the mid-point of the exponential phase. We then calculated T and S Ct values as the expected number of cycles required to reach the threshold. T- and S-values, i.e. the quantity of T and S products in each well, were then calculated from the slope and intercept of the standard curve, and the T/S ratios were finally calculated as T-value / S-value. We removed samples with Ct values >25 as they were deemed outliers and unreliable. We further calculated the relative difference in T/S ratio between duplicates and removed samples with relative difference >0.2, and averaged the T/S ratios of the remaining duplicates as the final measure of TL for each sample.

**Supporting tables & figures**

**Table S1.** Summary of the number of individuals with various numbers of samples in the Lundy house sparrow telomere dataset collected in 2000-2015

| *Number of samples* | *Number of individuals* |
| --- | --- |
| 1 | 749 |
| 2 | 256 |
| 3 | 126 |
| 4 | 53 |
| 5 | 22 |
| 6 | 14 |
| 7 | 3 |
| 8 | 1 |
| 9 | 1 |

**Table S2.** Summary of the number of birds and samples across age classes in the Lundy house sparrow telomere dataset collected in 2000-2015

| *Age in years* | *Number of birds* | *Number of samples* |
| --- | --- | --- |
| 0 | 703 | 800 |
| 1 | 535 | 669 |
| 2 | 248 | 298 |
| 3 | 144 | 175 |
| 4 | 64 | 78 |
| 5 | 35 | 40 |
| 6 | 15 | 16 |
| 7 | 5 | 7 |

**Table S3.** Summary of the full and pruned genetic pedigree of the Lundy house sparrows used in the telomere length analysis

| *Statistic* | *Full pedigree* | *Pruned pedigree* |
| --- | --- | --- |
| Records | 10731 | 1321 |
| Maternities | 8823 | 1196 |
| Paternities | 8951 | 1197 |
| Full sibs | 41647 | 1299 |
| Maternal sibs | 118459 | 3366 |
| Maternal half sibs | 76812 | 2067 |
| Paternal sibs | 132081 | 3553 |
| Paternal half sibs | 90434 | 2254 |
| Maternal grandmothers | 7933 | 1077 |
| Maternal grandfathers | 8130 | 1088 |
| Paternal grandmothers | 7862 | 1045 |
| Paternal grandfathers | 7887 | 1045 |
| Maximum pedigree depth | 20 | 16 |
| Founders | 1712 | 112 |
| Mean maternal sibship size | 13.74 | 3.60 |
| Mean paternal sibship size | 13.07 | 3.72 |
| Non-zero F | 6054 | 791 |
| F > 0.125 | 552 | 57 |
| Mean pairwise relatedness | 0.04 | 0.06 |
| Pairwise relatedness ≥ 0.125 | 0.10 | 0.14 |
| Pairwise relatedness ≥ 0.25 | 0.01 | 0.02 |
| Pairwise relatedness ≥ 0.5 | 0.001 | 0.004 |

**Table S4**. Summary of the full linear mixed model (LMM) testing for the telomere length–age relationship in the Lundy house sparrows sampled from 2000-2015, using parameter-expanded priors. Statistically significant estimates are in bold. Post. Mode = posterior mode, 95% CrI = 95% credible interval; pMCMC = MCMC p-value. WiAge = Within-individual age (in years); BtAge = between-individual age (in years); Blood Age = storage time as blood sample (in years); DNA Age = storage time as DNA sample (in years); Technician (N=2; contrast = A); BirdID = unique individual identifier; Plate = qPCR plate ID; Row = Row ID on qPCR plate; Units = residuals.

|  | *Post. mode* | *95% CrI* | *Effective sample size* | *pMCMC* |
| --- | --- | --- | --- | --- |
| Fixed effects | | | | |
| **(Intercept)** | **1.516** | **1.370 – 1.657** | **10374** | **<0.0001** |
| **WiAge** | **-0.056** | **-0.087 – -0.027** | **9000** | **<0.0001** |
| WiAge^2^ | -0.007 | -0.029 – 0.013 | 9000 | 0.463 |
| BtAge | 0.023 | -0.032 – 0.074 | 8915 | 0.454 |
| BtAge^2^ | 0.000 | -0.015 – 0.010 | 9000 | 0.759 |
| Sex | 0.005 | -0.040 – 0.052 | 9583 | 0.798 |
| **Blood Age** | **-0.095** | **-0.125 – -0.069** | **9732** | **<0.0001** |
| **Blood Age^2^** | **0.003** | **0.001 – 0.005** | **9775** | **<0.001** |
| DNA Age | 0.013 | -0.021 – 0.050 | 9000 | 0.412 |
| **DNA Age^2^** | **-0.006** | **-0.009 – -0.003** | **8709** | **<0.0001** |
| **Technician(B)** | **0.152** | **0.017 – 0.287** | **9524** | **0.027** |
|  | | | | |
| Random effects | | | | |
| BirdID | 0.036 | 0.022 – 0.051 | 9000 |  |
| Plate | 0.048 | 0.032 – 0.071 | 8255 |  |
| Row | 0.001 | 0.000 – 0.007 | 9000 |  |
| Units | 0.200 | 0.182 – 0.217 | 9000 |  |

**Table S5.** Summary of the random regression model (RRM) testing for among-individual variation in the mean telomere length (TL), and rate of TL change with age, among the Lundy house sparrows sampled in 2000-2015. Statistically significant estimates are in bold. Post. mode = posterior mode, 95% CrI = 95% credible interval; pMCMC = MCMC p-value. McAge = population mean-centred age; Blood Age = storage time as blood sample (in years); DNA Age = storage time as DNA sample (in years); Technician (N = 2; contrast = A); BirdID = unique individual identifier; Year = Year of capture; Plate = qPCR plate ID; Row = Row ID on qPCR plate; Units = residuals. DIC = 2683.

|  | | *Post. mode* | *95% CrI* | *Effective sample size* | *pMCMC* |
| --- | --- | --- | --- | --- | --- |
| Fixed effects | | | | | |
| **(Intercept)** | | **1.584** | **1.340 – 1.807** | **180000** | **<0.0001** |
| McAge | | -0.008 | -0.033 – 0.017 | 179123 | 0.861 |
| **Blood Age** | | **-0.103** | **-0.144 – -0.061** | **180000** | **<0.0001** |
| **Blood Age^2^** | | **0.003** | **0.001 – 0.006** | **180000** | **0.008** |
| DNA Age | | 0.019 | -0.030 – 0.070 | 180000 | 0.434 |
| **DNA Age^2^** | | **-0.007** | **-0.011 – -0.003** | **180000** | **0.001** |
| Technician (B) | | 0.018 | -0.114 – 0.147 | 180000 | 0.792 |
|  | |  |  |  |  |
| Random effects | | | | | |
| BirdID | |  |  |  |  |
|  | Intercept | 0.061 | 0.042 – 0.090 | 31670 |  |
|  | Slope | 0.081 | 0.052 – 0.124 | 28248 |  |
|  | **Intercept: Slope** | **0.030** | **0.011 – 0.061** | **26118** |  |
| Year | | 0.030 | 0.013 – 0.087 | 180000 |  |
| Plate | | 0.036 | 0.024 – 0.055 | 180000 |  |
| Row | | 0.001 | 0.000 – 0.008 | 180000 |  |
| Units | | 0.161 | 0.148 – 0.175 | 143582 |  |

**Table S6.** Individual variance-covariance matrix obtained from the character-state model fitted with a BirdID term only, built using telomere lengths from Lundy house sparrows sampled 2000-2015, pooled into age classes ‘juvenile (age 0)’, ‘young (ages 1-2)’, ‘middle-age (ages 3-4)’ and ‘old (ages 5 or above) within each individual. Additive genetic telomere length variance in each age class is shown on the diagonals, while inter-age-class covariances are shown on the off-diagonals. 95% credible intervals are shown in parentheses. Note none of the covariances were statistically significant. DIC = 1586.

|  | Juvenile | Young | Middle-age | Old |
| --- | --- | --- | --- | --- |
| Juvenile | 0.086  (0.090 – 0.152) |  |  |  |
| Young | 0.012  (-0.015 – 0.026) | 0.077  (0.072 – 0.117) |  |  |
| Middle-age | 0.003  (-0.038 – 0.030) | 0.003  (-0.032 – 0.030) | 0.155  (0.111 – 0.228) |  |
| Old | 0.007  (-0.043 – 0.044) | -0.002  (-0.040 – 0.034) | 0.004  (-0.055 – 0.067) | 0.240  (0.117 – 0.324) |

**Table S7.** Summary of the linear mixed model (LMM) testing for the linear telomere length–age relationship in the Lundy house sparrows sampled from 2000-2015, using inverse-Wishart priors. Statistically significant estimates are in bold. Post. mode = posterior mode, 95% CrI = 95% credible interval; pMCMC = MCMC p-value. WiAge = Within-individual age (in years); BtAge = between-individual age (in years); Blood Age = storage time as blood sample (in years); DNA Age = storage time as DNA sample (in years); Technician (N=2; contrast = A); BirdID = unique individual identifier; Plate = qPCR plate ID; Row = Row ID on qPCR plate; Units = residuals. N = 2078. DIC = 2876.

|  | *Post. mode* | *95% CrI* | *Effective sample size* | *pMCMC* |
| --- | --- | --- | --- | --- |
| Fixed effects | | | | |
| (Intercept) | 1.522 | 1.372 – 1.654 | 9000 | <0.0001 |
| **WiAge** | **-0.060** | **-0.086 – -0.026** | **9000** | **<0.0001** |
| BtAge | 0.008 | -0.011 – 0.032 | 9000 | 0.362 |
| Sex | 0.004 | -0.040 – 0.051 | 9000 | 0.850 |
| **Blood Age** | **-0.094** | **-0.126 – -0.068** | **9000** | **<0.0001** |
| **Blood Age^2^** | **0.003** | **0.001 – 0.005** | **9000** | **0.002** |
| DNA Age | 0.019 | -0.020 – 0.051 | 9319 | 0.400 |
| **DNA Age^2^** | **-0.006** | **-0.010 – -0.003** | **9484** | **<0.001** |
| **Technician(B)** | **0.143** | **0.014 – 0.284** | **9000** | **0.029** |
|  | | | | |
| Random effects | | | | |
| BirdID | 0.034 | 0.021 – 0.050 | 7567 |  |
| Plate | 0.044 | 0.031 – 0.070 | 9000 |  |
| Row | 0.001 | 0.000 – 0.007 | 9000 |  |
| Units | 0.200 | 0.182 – 0.216 | 9000 |  |

**Table S8.** Summary of the full linear mixed model (LMM) testing for the telomere length–age relationship in the Lundy house sparrows sampled from 2000-2015, assuming a relative within-individual ageing process, using inverse-Wishart priors. Statistically significant estimates are in bold. Post. Mode = posterior mode, 95% CrI = 95% credible interval; pMCMC = MCMC p-value. WiAge = Within-individual age (in years); BtAge = between-individual age (in years); Blood Age = storage time as blood sample (in years); DNA Age = storage time as DNA sample (in years); Technician (N=2; contrast = A); BirdID = unique individual identifier; Plate = qPCR plate ID; Row = Row ID on qPCR plate; Units = residuals. N = 2078. DIC = 2879.

|  | *Post. mode* | *95% CrI* | *Effective sample size* | *pMCMC* |
| --- | --- | --- | --- | --- |
| Fixed effects | | | | |
| (Intercept) | 1.498 | 1.367 – 1.654 | 9309 | <0.0001 |
| **WiAge** | **-0.056** | **-0.087 – -0.028** | **9564** | **<0.0001** |
| WiAge^2^ | -0.008 | -0.030 – 0.013 | 9000 | 0.439 |
| BtAge | 0.023 | -0.031 – 0.074 | 9000 | 0.415 |
| BtAge^2^ | -0.005 | -0.015 – 0.010 | 9000 | 0.715 |
| Sex | 0.008 | -0.041 – 0.051 | 9000 | 0.815 |
| **Blood Age** | **-0.096** | **-0.126 – -0.069** | **9000** | **<0.0001** |
| **Blood Age^2^** | **0.003** | **0.001 – 0.005** | **9000** | **0.001** |
| DNA Age | 0.015 | -0.021 – 0.051 | 9000 | 0.393 |
| **DNA Age^2^** | **-0.006** | **-0.010 – -0.003** | **9000** | **<0.001** |
| **Technician(B)** | **0.137** | **0.013 – 0.279** | **9000** | **0.028** |
|  | | | | |
| Random effects | | | | |
| BirdID | 0.036 | 0.022 – 0.050 | 8336 |  |
| Plate | 0.047 | 0.031 – 0.070 | 9000 |  |
| Row | 0.001 | 0.000 – 0.006 | 9000 |  |
| Units | 0.200 | 0.182 – 0.216 | 8734 |  |

**Table S9.** Summary of the full linear mixed model (LMM) testing for the telomere length–age relationship in the Lundy house sparrows sampled from 2000-2015, assuming an absolute within-individual ageing process, using inverse-Wishart priors. Statistically significant estimates are in bold. Post. Mode = posterior mode, 95% CrI = 95% credible interval; pMCMC = MCMC p-value. WiAge = Within-individual age (in years); BtAge = between-individual age (in years); Blood Age = storage time as blood sample (in years); DNA Age = storage time as DNA sample (in years); Technician (N=2; contrast = A); BirdID = unique individual identifier; Plate = qPCR plate ID; Row = Row ID on qPCR plate; Units = residuals. N = 2078. DIC = 2874.

|  | *Post. mode* | *95% CrI* | *Effective sample size* | *pMCMC* |
| --- | --- | --- | --- | --- |
| Fixed effects | | | | |
| (Intercept) | 1.530 | 1.367 – 1.654 | 9000 | <0.0001 |
| **WiAge** | **-0.104** | **-0.173 – -0.050** | **9000** | **<0.0001** |
| (Age^2^ – BtAge^2^) | 0.012 | -0.000 – 0.023 | 9000 | 0.050 |
| BtAge | 0.017 | -0.043 – 0.062 | 9000 | 0.696 |
| BtAge^2^ | -0.001 | -0.014 – 0.011 | 9000 | 0.833 |
| Sex | 0.006 | -0.041 – 0.050 | 9000 | 0.838 |
| **Blood Age** | **-0.104** | **-0.130 – -0.073** | **9000** | **<0.0001** |
| **Blood Age^2^** | **0.003** | **0.001 – 0.005** | **9000** | **0.001** |
| DNA Age | 0.015 | -0.020 – 0.052 | 8006 | 0.370 |
| **DNA Age^2^** | **-0.007** | **-0.010 – -0.003** | **9000** | **<0.001** |
| **Technician(B)** | **0.125** | **0.013 – 0.276** | **9000** | **0.032** |
|  | | | | |
| Random effects | | | | |
| BirdID | 0.035 | 0.022 – 0.051 | 8196 |  |
| Plate | 0.044 | 0.030 – 0.068 | 9000 |  |
| Row | 0.001 | 0.000 – 0.007 | 9000 |  |
| Units | 0.198 | 0.181 – 0.215 | 8843 |  |

**Table S10**. Random effect structures, individual repeatability (R), heritability (h^2^) and DICs from a series of ‘animal’ models estimating sources of variation in telomere length in the Lundy house sparrows sampled in 2000-2015. We report here the posterior modes and 95% credible intervals of estimates. Animal = Additive genetic variance estimated from genetic pedigree; Bird ID = permanent environmental variance; Rearing Mum ID = identity of rearing mother; Rearing Dad ID = identity of rearing father; Year = Year of sampling; Cohort = Year when an individual was born; Plate ID = qPCR plate identity; Row ID = row where sample was located on the qPCR plate. Plate and row variance were included in the models but excluded when calculating R and h^2^.

| *Model* | *Animal* | *Bird ID* | *Rearing Mum ID* | *Rearing Dad ID* | *Year* | *Cohort* | *Plate ID* | *Row ID* | *Residual* | *R* | *h^2^* | *DIC* |
| --- | --- | --- | --- | --- | --- | --- | --- | --- | --- | --- | --- | --- |
| 3 | 0.037 (0.023 – 0.053) | 0.000  (0.000 – 0.015) | / | / | / | / | 0.046 (0.030 – 0.070) | 0.001 (0.000 – 0.008) | 0.196 (0.181 – 0.211) | 0.182  (0.125 – 0.237) | 0.151  (0.101 – 0.216) | 2807 |
| 4 | 0.038  (0.023 – 0.053) | 0.000  (0.000 – 0.015) | 0.000  (0.000 – 0.002) | / | / | / | 0.046 (0.031 – 0.070) | 0.001 (0.000 – 0.007) | 0.194 (0.181 – 0.211) | 0.184  (0.123 – 0.235) | 0.153  (0.099 – 0.215) | 2809 |
| 5 | 0.037  (0.023 – 0.053) | 0.000  (0.000 – 0.015) | 0.000  (0.000 – 0.002) | 0.000  (0.000 – 0.002) | / | / | 0.045 (0.031 – 0.070) | 0.001 (0.000 – 0.008) | 0.195 (0.180 – 0.211) | 0.177  (0.123 – 0.235) | 0.152  (0.100 – 0.216) | 2810 |
| 6 | 0.033  (0.020 – 0.048) | 0.000  (0.000 – 0.015) | 0.000  (0.000 – 0.002) | 0.000  (0.000 – 0.002) | 0.031  (0.013 – 0.094) | / | 0.037 (0.025 – 0.056) | 0.002 (0.000 – 0.010) | 0.178 (0.165 – 0.194) | 0.144  (0.093 – 0.201) | 0.126  (0.074 – 0.182) | 2641 |
| 7 | 0.031  (0.020 – 0.048) | 0.000  (0.000 – 0.014) | 0.000  (0.000 – 0.002) | 0.000  (0.000 – 0.002) | 0.031  (0.012 – 0.090) | 0.000  (0.000 – 0.005) | 0.037 (0.025 – 0.056) | 0.001 (0.000 – 0.010) | 0.181 (0.165 – 0.194) | 0.140  (0.091 – 0.199) | 0.123  (0.075 – 0.182) | 2641 |

**Table S11.** Inter-age variance-covariance matrix showing the age-related structure of additive genetic variance in telomere length in the Lundy house sparrows sampled in 2000-2015. Estimates (and 95% CrIs) for a total of eight age classes (Age 0 to Age 7) were obtained from back-transformation of random regression ‘animal’ model (RRAM) coefficients. Within age-class variances are shown on the diagonal while inter-age-class covariances are shown on the off-diagonal. Significant covariances are in bold. Note that due to back-transformation, some estimates could lie outside of their CrIs. We treated these estimates as not statistically significant.

|  | *Age 0* | *Age 1* | *Age 2* | *Age 3* | *Age 4* | *Age 5* | *Age 6* | *Age 7* |
| --- | --- | --- | --- | --- | --- | --- | --- | --- |
| *Age 0* | 0.100  (0.077 - 0.116) |  |  |  |  |  |  |  |
| *Age 1* | **0.073**  **(0.058 - 0.082)** | 0.056  (0.046 - 0.061) |  |  |  |  |  |  |
| *Age 2* | **0.047**  **(0.040 - 0.047)** | **0.039**  **(0.034 - 0.041)** | 0.031  (0.027 - 0.034) |  |  |  |  |  |
| *Age 3* | **0.020**  **(0.013 - 0.022)** | 0.022  (0.020 - 0.021) | **0.023**  **(0.021 - 0.027)** | 0.024  (0.020 - 0.034) |  |  |  |  |
| *Age 4* | **-0.006**  **(-0.022 - 0.004)** | 0.004  (-0.001 - 0.009) | **0.015**  **(0.014 - 0.020)** | **0.026**  **(0.019 - 0.041)** | 0.036  (0.024 - 0.062) |  |  |  |
| *Age 5* | **-0.032**  **(-0.056 - -0.014)** | **-0.013**  **(-0.021 - -0.003)** | 0.007  (0.008 - 0.014) | **0.027**  **(0.019 - 0.048)** | **0.047**  **(0.030 - 0.083)** | 0.066  (0.041 - 0.118) |  |  |
| *Age 6* | **-0.059**  **(-0.09 - -0.032)** | **-0.030**  **(-0.042 - -0.016)** | -0.001  (0.001 – 0.007) | **0.028**  **(0.018 - 0.056)** | **0.057**  **(0.035 - 0.104)** | **0.086**  **(0.052 - 0.153)** | 0.115  (0.068 - 0.201) |  |
| *Age 7* | **-0.085**  **(-0.125 - -0.051)** | **-0.047**  **(-0.062 - -0.028)** | -0.009  (-0.005 – 0.000) | **0.029**  **(0.017 - 0.063)** | **0.068**  **(0.040 - 0.125)** | **0.106**  **(0.063 - 0.188)** | **0.144**  **(0.085 - 0.250)** | 0.182  (0.108 - 0.313) |

**Table S12.** Genetic and permanent environmental variance-covariance matrices obtained from the character state model, built using telomere lengths from Lundy house sparrows sampled 2000-2015, pooled into stages ‘juvenile (age 0)’, ‘young (ages 1-2)’, ‘middle-age (ages 3-4)’ and ‘old (ages 5 or above) within each individual. Telomere length variance in each state is shown on the diagonals, while inter-stage covariances are shown on the off-diagonals. 95% credible intervals are shown in parentheses. Note none of the covariances were statistically significant. DIC = 1495.

|  | Juvenile | Young | Middle-age | Old |
| --- | --- | --- | --- | --- |
| Additive genetic | | | | |
| Juvenile | 0.086  (0.065 – 0.114) |  |  |  |
| Young | 0.012  (-0.004 – 0.027) | 0.077  (0.060 – 0.102) |  |  |
| Middle-age | 0.003  (-0.029 – 0.030) | 0.003  (-0.024 – 0.029) | 0.155  (0.102 – 0.228) |  |
| Old | 0.007  (-0.038 – 0.044) | -0.002  (-0.037 – 0.040) | 0.004  (-0.066 – 0.075) | 0.240  (0.136 – 0.441) |
|  | | | | |
| Permanent environmental | | | | |
| Juvenile | 0.094  (0.074 – 0.124) |  |  |  |
| Young | 0.000  (-0.016 – 0.016) | 0.073  (0.060 – 0.095) |  |  |
| Middle-age | -0.000  (-0.029 – 0.026) | 0.003  (-0.023 – 0.025) | 0.144  (0.101 – 0.210) |  |
| Old | 0.000  (-0.044 – 0.040) | -0.006  (-0.035 – 0.032) | 0.000  (-0.064 – 0.063) | 0.233  (0.132 – 0.409) |

**
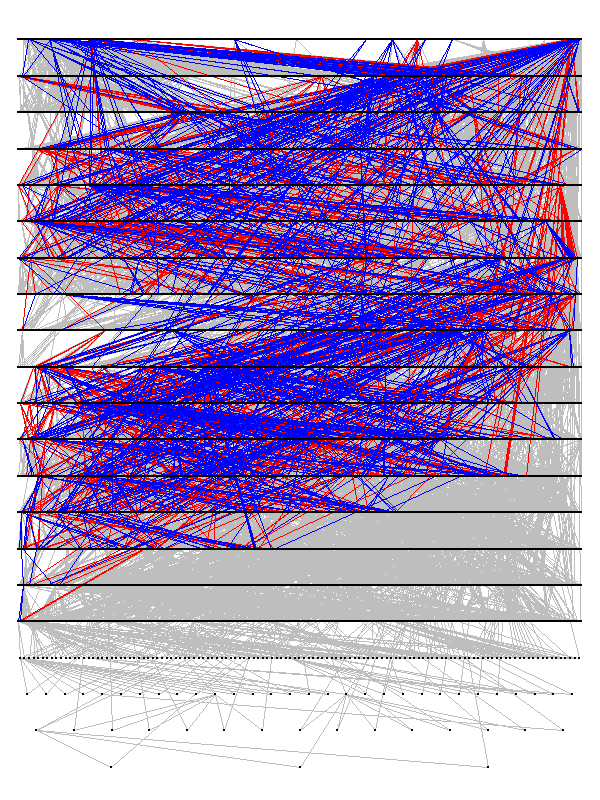
**

**Fig. S1.** The pruned pedigree of the Lundy House Sparrows used to estimate heritability of telomere length and the rate of telomere shortening. Each dot represents one individual, red lines represent maternities, blue lines represent paternities, and grey lines represent links with non-phenotyped but informative individuals.

**SI References**

Cawthon, R. M. (2002). Telomere measurement by quantitative PCR. *Nucleic Acids Research*, *30*(10), 1–6. https://doi.org/10.1093/nar/30.10.e47

Cawthon, R. M. (2009). Telomere length measurement by a novel monochrome multiplex quantitative PCR method. *Nucleic Acids Research*, *37*(3), e21–e21. https://doi.org/10.1093/nar/gkn1027

Sibma, A. (2021). *A longitudinal analysis of telomeres in an insular house sparrow population* [PhD thesis]. University of Sheffield.

van Lieshout, S. H. J., Froy, H., Schroeder, J., Burke, T., Simons, M. J. P., & Dugdale, H. L. (2020). Slicing: A sustainable approach to structuring samples for analysis in long‐term studies. *Methods in Ecology and Evolution*, *11*(3), 418–430. https://doi.org/10.1111/2041-210X.13352
